# Delayed Pulmonary Oedema Following Attempted Suicidal Hanging–A Case Report

**Published:** 2009-06

**Authors:** Mahendra Kumar, Rajani Mandhyan, Usha Shukla, Ashok Kumar, R S Rautela

**Affiliations:** 1,4,5 Professor, Dept. of Anaesthesiology and Critical Care, University College of Medical Sciences and GTB Hospital, Shahdara Delhi 110095; 2,3 Senior Resident, Dept. of Anaesthesiology and Critical Care, University College of Medical Sciences and GTB Hospital, Shahdara Delhi 110095

**Keywords:** Suicidal hanging, Hypoxia, Pulmonary oedema, Raised intra cranial pressure, Convulsions

## Abstract

**Summary:**

During suicidal hanging, death takes few minutes to occur. Patient, if rescued, may develop respiratory distress, pulmonary oedema, convulsions, raised intra cranial pressure and unconsciousness immediately after incidence. We report a young male of suicidal hanging, brought to hospital in unconscious state with decerebrating movements. He developed pulmonary oedema after two hours of incidence. He was resuscitated and treated successfully.

## Introduction

Hanging is a common mode of committing suicide with a high incidence among the suicidal cases.^1^,^2^ The death usually occurs within few minutes of hanging.^3^ Most of the patients develop respiratory and neurological complications immediately after incidence. Pulmonary oedema one of the most common complications occurs in patients immediately following their rescue from acute airway obstruction or suicidal hanging.^4^–^6^ We report a case of suicidal hanging who developed pulmonary oedema that was delayed by two hours following incidence. He presented in unconsciousness state with decerebrating movements. Intensive therapy was directed towards improvement in oxygenation, reduction in raised intra cranial pressure (ICP) and prevention of neurological consequences by cerebral resuscitation.

## Case report

A 25-year-old male patient was admitted to intensive care unit in unconscious state with an alleged history of suicidal hanging by neck. Patient was rescued by his relatives within few minutes and shifted to hospital within 30 minutes. On examination his pulse rate was 126/min and arterial blood pressure was 90/64 mmHg. His pupils were bilaterally dilated and nonreacting to light. On auscultation chest was bilaterally clear with adequate air entry, but patient was tachypnoeic with respiratory rate of 32/min. Upper airway reflexes were present. Patient had intermittent decerebrating movements. Pulse oximetry revealed SpO_2_ 60% and arterial blood gas (ABG) analysis showed PaO_2_ 54mmHg, PaCO_2_ 28mmHg, pH 7.26, BE 9.2mEq/L and SaO_2_ 64% on air.

Immediately, his trachea was intubated orally with 8.5 mm ID cuffed endotracheal tube after thiopentone sodium 200 mg and succinylcholine 75 mg I.V. Patient was put on ICU ventilator for controlled ventilation with respiratory rate of 12/min, tidal volume 500mL, PEEP 5cm of H_2_O and FiO_2_ 1.0. Patient was given pancuronium and midazolam. After one and half hour of therapy, SpO_2_ further dropped to 52% and patient developed frank pulmonary oedema with extensive fine crepts all over the chest and pink frothy secretions appeared in the breathing system. Controlled ventilation was continued with PEEP. He was given furosemide 60 mg and morphine 6 mg IV and repeated 6 hourly. X-rays of cervical spine AP and lateral view revealed no bony injury. After 2hours patient's ABG was satisfactory and revealed PaO_2_94mmHg, PaCO_2_29mmHg, SaO_2_94% on FiO_2_1. His chest condition was markedly improved after 12 hours of ventilation. His ABG analysis depicted PaO_2_114mmHg, PaCO_2_35mmHg, pH 7.41, SaO_2_98.4% on FiO_2_ 0.5. He regained his consciousness. He was put on weaning mode of ventilation Assisted Spontaneous Breath(ASB) and Continuous Positive Airway Pressure(CPAP) with FiO_2_ 0.4 for next 12 hrs. After 24 hours (2^nd^ day) he was fully conscious. His SpO_2_ was 99% on FiO_2_ 0.4. Chest X-ray AP view detected no abnormality. ABG analysis revealed PaO_2_ 116mmHg, PaCO_2_ 45mmHg, pH 7.49 and SaO_2_ 99% on FiO_2_ 0.4. On 3^rd^ day his trachea was extubated Post extubation his SpO_2_ was 97% on FiO_2_ 0.4 by facemask.

## Discussion

Hanging is a very common mode of suicide particularly in young adults.^1^ Its incidence in India is approximately 25% of total cases of suicide.^2^

In judicial hanging death is instantaneous due to fall of body for few meters in the air, causing fracture and/or dislocation of cervical vertebrae and vasovagal shock.^3^ However, in suicidal hanging these injuries are rare and death is often a slow process, which takes about 8-10 min. Death in suicidal hanging is secondary to hypoxia and cerebral ischaemia due to compression of airway and major blood vessels of neck caused by ligature applied round the neck and the force of compression being the body weight.^3^ If patient is rescued within few minutes of hanging, may be saved by applying specific resuscitative measurements.

The clinical features of a patient of hanging involve respiratory and central nervous system. The common respiratory signs are respiratory distress, hypoxia, pulmonary oedema etc; and signs related to CNS are like restlessness, unconsciousness, muscular rigidity, convulsions, amnesia, hemiplegia etc.^3^

Our patient had unconsciousness, muscular rigidity and decerebrating movements, respiratory distress and hypoxia at the time of admission. He developed pulmonary oedema two hours later during controlled ventilation through endotracheal tube.

Pulmonary oedema has been reported in literature following a sudden relief from upper airway obstruction.^4^–^6^ Its onset is very rapid, generally appears within minutes of the event but some times it may be delayed. The cause of delay is not clear but it might be related to rate of onset of oedema and severity of airway obstruction.^6^ The exact mechanism of development of pulmonary oedema after rescue from hanging or strangulation is still not clear. Some workers postulate that cerebral hypoxia during hanging causes release of vasoactive substances like histamine, serotonin and kinins. These mediators along with hypoxia lead to pulmonary vasoconstriction, pulmonary hypertension and pulmonary congestion.^4^,^7^,^8^ Second theory suggests that pulmonary capillary membrane is damaged leading to increased capillary permeability and hence pulmonary oedema.^4^ Third theory suggests that the cause of pulmonary oedema is hyperaemia in the lungs. If airway obstruction is suddenly removed there is an abrupt fall in intrapulmonary pressure, which suddenly increases the venous return and hence increases pulmonary hyperaemia.^8^

Presentation of patient with accumulated fluid in lungs may range from hyperaemia to frank pulmonary oedema through lung congestion. Perhaps, our patient was in the state of hyperaemia in the lungs at the time of admission as he was showing hypoxia without any added sound in the chest. Later on he developed frank pulmonary oedema, it might be because of continuous process of accumulation of fluid in the lungs either under influence of vasoactive substances or damaged pulmonary capillary membrane. Thus, any patient having hypoxia following rescue from hanging or relief from upper airway obstruction with clear chest may be considered as a case of hyperaemia and such patient may develop delayed frank pulmonary oedema during therapy.

Airway obstruction and compression of blood vessels in neck causes cerebral oedema, hypoxic insult, raised intra cranial pressure (ICP) and neurological manifestations.

The strategy of management should aim to get adequate oxygenation and cerebral perfusion.^4^ In presence of respiratory distress (hypoxia) with or without pulmonary oedema tracheal intubation and mechanical ventilation are indicated. PEEP (positive end expiratory pressure) has its definite role in the treatment of pulmonary oedema, but at the same time it also increases intracranial pressure, so one has to weigh its use according to patient's condition.^9^–^11^ Hyperventilation is beneficial to the patient having raised ICP for short term therapy.^10^

To ensure adequate cerebral perfusion, immediate steps should be taken to reduce raised ICP by using hyperventilation and diuretics eg frusemide.^4^ Fluid overloading and mannitol should be avoided even in the absence of lung oedema. If use of mannitol is mandatory, it should be used along with frusemide to avoid over expansion of plasma volume. Morbidity is the result of post obstruction effects. After rescue from airway obstruction, reestablishment of patent airway may not provide the proper oxygenation even with the clear chest as occurred in our patient. Such patient may require aggressive oxygen therapy for adequate oxygenation. Pulmonary oedema is reversible in most of the cases once recognized and treated properly. Although neurological injury determines the outcome, but poor initial condition does not exclude a good recovery.^12^ To conclude, patients rescued from suicidal hanging or strangulation even with clear chest must be treated with aggressive oxygen therapy and monitored closely as they may develop pulmonary oedema even after few hours following the event.
